# Antimicrobial resistance: a top ten global public health threat

**DOI:** 10.1016/j.eclinm.2021.101221

**Published:** 2021-11-24

**Authors:** EClinicalMedicine

Health crises such as viral pandemics arise suddenly and require immediate actions and responses; others come to light more slowly and are more inconspicuous and intractable. An example of the latter is antimicrobial resistance (AMR), which was reported shortly after the introduction of the first effective antimicrobial agent and has been declared as one of the top ten global public health threats facing humanity by WHO in 2019.

AMR occurs when microbes, such as bacteria, viruses, fungi, and parasites, adapt over time and no longer respond to drugs to which they were initially sensitive, making infections harder to treat and resulting in an increased risk of disease spread, and severe illness and death following routine medical procedures such as surgery. In 2019, the Interagency Coordination Group on Antimicrobial Resistance estimated that drug-resistant diseases are responsible for at least 700 000 deaths globally per year, a figure that could potentially increase to 10 million deaths globally per year by 2050.

Although AMR is a natural phenomenon, resistance develops more rapidly through the misuse and overuse of antimicrobial agents. Therefore, health-care workers play a pivotal role in preventing AMR. The English Surveillance Programme for Antimicrobial Utilisation and Resistance Report 2019 to 2020, published by Public Health England, noted that the total consumption of antibiotics in England decreased by 7•5% between 2015 and 2019. This decrease is likely attributable to an increased awareness of AMR by health-care professionals, as well as due to the effect of government monetary incentives such as the Quality Premiumscheme, which was introduced in 2015 to reward general practitioners for improvements in quality of care, including the reduction of inappropriate antibiotic prescribing in primary care. However, hospitals remain a key site for the transmission of antimicrobial-resistant bacteria, emphasising the importance of good hygiene and infection prevention and control protocols as well as evidence-based consideration of the appropriate prescribing of antimicrobials. Hospital inpatients are therefore at an increased risk of developing AMR compared with outpatients. Of note, according to WHO, people with a methicillin-resistant *Staphylococcus aureus* infection, which is primarily a hospital-acquired infection, are 64% more likely to die than people with drug-sensitive infections.

While the impact of AMR on human health has been widely documented, the misuse or overuse of antimicrobial agents in agriculture and farming is also a substantial concern. For farmers and the food industry, a lack of effective antimicrobial agents to treat sick animals adversely affects food production. Farmers face an additional risk due to their close contact with animals that might be colonised or infected with resistant bacteria. Furthermore, resistant bacteria from animals can be transferred to humans via the consumption of animal products that contain antibiotic-resistant bacteria. Another risk for human infection with resistant organisms can arise from the consumption of vegetables that have been treated with antimicrobial agents. While AMR in agriculture and farming poses a significant risk to human health and economic wellbeing, the Farming and Agriculture Organisation of the UN reports that only 118 countries collected data on the use of antimicrobials in animals between 2015 and 2017, and notably fewer produce data for plant agriculture, highlighting the need for improved reporting in this area.

To try and address some of these concerns, the WHO Global Action Plan on AMR was launched in 2015 with the intention of optimising the use of antimicrobial agents, and to attract investment in the research and development of new agents; an area which has stalled in recent decades. The action plan calls for initiatives to be accessible in an affordable and equitable way to those in low-resource settings. The plan also highlights the need for an effective one health approach, involving multipronged initiatives and policies across human and veterinary medicine, agriculture, and the environment.

The WHO Global Antimicrobial Resistance and Use Surveillance System (GLASS), was introduced in 2015 to help accomplish the objectives of the WHO Global Action Plan on AMR, and promotes the standardisation of the collection and sharing of AMR surveillance data across the globe. The first GLASS report, published in 2018, included data on more than 500 000 infections from 22 countries. Since then, the number of countries participating in GLASS has increased to 109, of which 107 provide AMR data, and 17 measure antibiotic consumption. Setting up country-level surveillance of resistance and consumption is vital for understanding the impact of AMR and to reduce the spread of resistant pathogens. Another initiative is World Antimicrobial Awareness Week, which was celebrated on Nov 18–24, 2021, and is a yearly event organised by the Food and Agriculture Organization of the UN, the World Organisation for Animal Health, and WHO. The event aims to promote awareness on antimicrobial resistance and to prevent further spread of drug-resistant infections.

Despite increased participation in WHO-led initiatives to combat AMR, and improvement in AMR surveillance systems in several countries, further AMR data are required to identify interventions that might slow resistance and to determine the effect of AMR on human health in different countries. Collaborative efforts between doctors, veterinarians, industry, and regulators are required, and all stakeholders should be aware of their vital role in preserving the effectiveness of antimicrobial agents so that these agents can be targeted to diseases where their use is appropriate. Treatment should also be stopped when no longer indicated. These efforts will help to ensure effective drugs are not lost from future patient management.

